# Machine learning in optimization of nonwoven fabric bending rigidity in spunlace production line

**DOI:** 10.1038/s41598-023-44571-z

**Published:** 2023-10-17

**Authors:** Mohammad Reza Sadeghi, Seyed Mohammad Hosseini Varkiyani, Ali Asghar Asgharian Jeddi

**Affiliations:** Department of Textile Engineering, Amirkabir University of Technology, Tehran, Iran

**Keywords:** Engineering, Mathematics and computing

## Abstract

Spunlace nonwoven fabrics have been extensively employed in different applications such as medical, hygienic, and industrial due to their drapeability, soft handle, low cost, and uniform appearance. To manufacture a spunlace nonwoven fabric with desirable properties, production parameters play an important role. Moreover, the relationship between the primary response and input parameter and the relationship between the secondary response and primary responses of spunlace nonwoven fabric were modeled via an artificial neural network (ANN). Furthermore, a multi-objective optimization via genetic algorithm (GA) to find a combination of production parameters to fabricate a sample with the highest bending rigidity and lowest basis weight was carried out. The results of optimization showed that the cost value of the best sample is 0.373. The optimized set of production factors were Young’s modulus of fiber of 0.4195 GPa, the line speed of 53.91 m/min, the average pressure of water jet 42.43 bar, and the feed rate of 219.67 kg/h, which resulted in bending rigidity of 1.43 mN ${\mathrm{cm}}^{2}$/cm and basis weight of 37.5 gsm. In terms of advancing the textile industry, it is hoped that this work provides insight into engineering the final properties of spunlace nonwoven fabric via the implementation of machine learning.

## Introduction

Bending rigidity is one of the important characteristics of fabric that affects the final consumption of fabric and clothing such as drape, buckling, hand, comfort, formability, stitchability, and wrinkle^1–3^. Woven and knitted fabrics exhibit low bending rigidity due to the relatively free movement of fibers and yarns. However, nonwoven fabrics possess higher bending rigidity for equivalent basis weights, showcasing their structural stability and firmness^4–7^. In the quest for more efficient fabric production for apparel, researchers and companies are exploring streamlined methods to reduce time and costs while maintaining quality^8^. Generally, in fashion apparel and industrial applications, low and high bending rigidity are preferred respectively^2,3,9^. Nonwoven fabrics can be defined as structures produced by bonding or the interlocking of randomly dispersed fibers or filaments, excluding paper that are bonded or entangled by mechanical, thermal or chemical means and may be modified by additional chemical treatments referred to as finishing^10,11^. Nonwovens refer to materials that are not produced through weaving, knitting, tufting, or wool felting processes^11^. Flame retardancy, Absorbency, Softness, washability, Filtering, Bacterial barrier, and Liquid repellency can be mentioned among the characteristics of nonwoven fabrics^12^. Nonwoven textiles, also known as engineered fabrics, are becoming increasingly popular in various industries due to their faster production rate, wider availability, and lower cost compared to traditional woven and knitted fabrics. As a result, nonwoven fabrics are replacing traditional fabrics in many applications^13^. To produce nonwoven fabrics, several steps are involved, including web formation, web bonding, and web finishing. The bonding process involves techniques such as needle punching, hydro-entangling, thermal, chemical, and adhesive bonding to create a nonwoven fabric by bonding the webs together. Hydro-entangling is a mechanical bonding process that aims to produce nonwoven fabrics with a texture and appearance similar to woven and knitted fabrics^13,14^. The main energy used in the spunlace method is water energy^10,15–18^. Its similar names are water-jet entangled nonwovens, hydro-entangled nonwovens, hydro-entangled fabrics (HEF), and hydraulically needled fabrics^19^. Hydro-entanglement technology has been developed for more than forty years and in the nonwovens industry, the hydro-entanglement process is the fastest growing technology^8,13,20–23^. Roughly 12% of the global production of nonwovens is believed to be created using the hydro-entanglement technique. Fabrics produced through hydro-entanglement possess unique characteristics, including a soft and flexible texture, high drape, excellent absorbency, high bulk, comfortable feel, low linting, stretchable without losing thickness, high strength without binders, and resistance to delamination^21,24^. Hydro-entangled nonwovens are strong, soft and flexible depending on the fibers constituent and can be dense or open and are generally absorbent. As a result of these properties, hydro-entangled fabrics are commonly utilized in the medical, personal care, baby care, consumer, and hygiene markets, especially for fine fiber webs^21^. Among the major applications of spunlace nonwoven textiles, can be mentioned medical/surgical, wipes, home furnishings (mattress pad covers, table cloths, bedspreads), disposable apparel (lab coats), substrates (coated backing, reinforce plastic), insulation, roofing, molded products, interlining, artificial leather, geotextiles, automotive parts, apparel, furnishings, filtration, and hygiene products^13,19^. The application of nonwoven textiles is progressing. The main difference between woven and nonwoven fabrics is their bending rigidity, so that the bending rigidity is higher in nonwoven fabrics. This is due to the different mechanical principles of deformations in the two structures^11^.

Due to the best abilities of the human brain in teaching and learning various issues, intelligent systems have a structure similar to the human brain has been the focus of researchers for many years. One of these systems is called an artificial neural network. Artificial neural networks and simpler terms neural networks known by names such as parallel distributed processing models, communication models, and neuromorphic systems^25^. Neural networks are simplified processing units resembling the neurons in human and animal brains. Through the adjustment and matching of these units via teaching patterns, they demonstrate the ability to process information and learn. Neural networks find extensive applications in solving diverse scientific, engineering, and commercial problems, offering a powerful tool for tackling complexity^26,27^. An artificial neural network (ANN) is an intelligent system that has many applications in research and various engineering fields^28–32^. The most important applications of neural networks in the textile are the prediction of some output data from input data, identification of unknown principles between data (classification), detection of data containing errors and batch communication of input data, and finding noise of input data. Artificial neural networks have been used in many fields of engineering to predict the properties of materials, and many applications have been reported in the textile industry, especially on fabrics^33–37^. Here, the studies conducted on the ANN in the field of mechanical properties, especially the bending, are briefly presented. Behera and Guruprasad reported research on the ability to predict the bending properties of woven fabrics from their structure using the ANN method. Yarn density, yarn count, yarn twist, and weave type were considered as input parameters, and bending rigidity in the direction of warp and weft was considered as output. The total prediction error of the model was 80.7%, which indicates a good prediction of developed model. Also, a good correlation has been obtained between the actual values and the predicted values with R^2^ = 0.99^38^. Using a neural network system, Chenganmal considered the fabric parameters such as yarn count, number of wefts (ends/cm), filament denier, and weight thickness as input data. The output results showed the bending rigidity with a high correlation coefficient value of R^2^ = 0.90. This research showed that the prediction of the bending rigidity of plain fabrics was very satisfactory. The proposed method was very useful for determining the bending rigidity in the clothing industry. The correlation coefficient of bending rigidity between KESF (Kawabata evaluation system of fabric) values and predicted values was 0.97^39^. Debnath et al. studied the tensile properties of needle-punched nonwoven fabrics made from a blend of polypropylene and jute fibers. They developed experimental models and artificial neural networks to predict the tensile properties and compared the results with experimental values. By employing a back-propagation algorithm for a hidden multi-layer network, they achieved reliable predictions for the tensile properties of needle-punched nonwoven fabrics^34,40^. Semnani et al. showed that ANN can be used to predict the appearance parameters of fabric to evaluate its appearance quality very accurately and correctly^41^. Elkateb presented a model to accurately predict the mechanical properties of woven fabric based on ANN. The results indicate that ANN has excellent performance in predicting all features^42^. Jin and Zhu investigated a model to predict the pore size and its distribution for melt blown nonwovens. They found that the back-propagation model predicts much more strongly than the multiple linear regression model and the error percentage of this method was less than 5% in all samples^43^. Chen et al. predicted the fiber diameter of melt blown with the ANN model. They concluded that the ANN model is very accurate and calculated its average error of 0.013%^44^. They showed that the ANN model can provide accurate and reasonably good predictions with relatively few data to model structure–property relations of nonwoven fabrics for filtration applications^45^. Rawal et al. predicted the properties of needle-punched nonwoven using ANN, and the correlation between experimental and predicted values was good, although the number of samples was limited^36^.

Genetic algorithms (GA) are search algorithms inspired by natural selection and genetics. They employ principles from evolutionary biology, such as inheritance, mutation, selection, and crossover. GA is a global search heuristic used to find optimal or approximate solutions for optimization and search problems. It is widely utilized in various research fields and has become an advanced and powerful tool for engineering optimization and solving numerical problems^46^. Dubrovski and Brezocnik employed modeling techniques to assess the porous properties of untreated polyester/viscose nonwoven wipes. Their analysis considered nonwoven construction parameters like fiber fineness, web mass per unit area, and web thickness, along with mercury intrusion porosimetry and genetic algorithms. The results demonstrated that genetic algorithms proved effective as a modeling tool, especially with limited sample sizes^47^. They also utilized genetic algorithms to develop a model that could predict the water absorption capacity of needle-punched webs. Their analysis revealed that the proposed model was capable of accurately predicting the desired water absorption capacity^48^. In another work, they used the GA method to study the wicking rate of needle-punched nonwoven, and the results were in good agreement with experimental ones^49^. Han and Wang designed the optimal coat-hanger die used for in melt blown nonwoven using GA. It was shown that the systematic approach combining the application of numerical simulation and GA is an effective way to optimize the geometry parameters of the coat-hanger die^50^.

This work aims to find an optimal set of factors for the production of spunlace using different procedures of modeling technique consisting of ANN and response surface methodology (RSM) and GA as an optimizer, in which it leads to highest bending rigidity and lowest basis weight for rug industry. For the development of the model, an experiment based on the central composite design of experiment (CCDOE) was conducted. To verify the validity of the proposed hybrid method, the optimized sample is fabricated and compared with the predicted value.

## Modeling

Before the modeling of either response surface methodology (RSM) or artificial neural network, the repetition values were averaged, and the final data were normalized between 0.1 and 0.9 to avoid the quantitative effect.

### Response surface methodology

Among the different regression models, RSM is mostly used to develop the relationship between independent and dependent variables. Such a method is capable of constructing a multivariate non-linear formula with acceptable precision of prediction. Using experimental data, the coefficients and offset values are determined^51–54^. In this study, two sets of response parameters including primary and secondary based on the physics of the problem were considered. The primary responses are basis weight, thickness, number of couples, and fiber orientation angle in the MD direction. The input parameters of the formula developed for primary responses are Young’s modulus, line speed, the average pressure of the water jet, and feed rate. Equation (1) represents the general formula used for the four mentioned primary responses.

1 $$\widehat{f}={a}_{0}+\sum_{1}^{4}{a}_{i}{x}_{i}+\sum_{1}^{4}\sum_{1}^{4}{a}_{ij}{x}_{i}{x}_{j}+\sum_{1}^{4}{a}_{ii}{x}_{i}^{2},$$

where ${a}_{0}$, ${a}_{i}$, ${a}_{ij}$, and ${a}_{ii}$ are offset, the linear effect of the corresponding parameter, the linear–linear interaction effect of parameters, and the quadratic effect of the corresponding parameter, respectively. The second developed function which is called secondary response is represented by Eq. (2), where four primary responses are fed as input parameters.

2 $$\widehat{g}={a}_{0}+\sum_{1}^{7}{a}_{i}{x}_{i}+\sum_{1}^{7}\sum_{1}^{7}{a}_{ij}{x}_{i}{x}_{j}+\sum_{1}^{7}{a}_{ii}{x}_{i}^{2}.$$

To determine the coefficients and offset values of models, the same procedure as^55,56^ was employed under Excel software.

### Artificial neural network

Nowadays, artificial neural network (ANN) as an advanced procedure of modeling is enormously applied in the engineering of materials and processes. Furthermore, the fitness of artificial neural networks is much higher than regression models if a complex relationship between variables exists. Using a feed-forward back-propagation learning algorithm, any function with any degree of complexity can be estimated. The precision of such networks is increased when a single output is considered^57–63^. In this work, four parallel feed-forward back-propagation networks for primary responses including basis weight ($W$), thickness ($T$), number of couples ($C$), and fiber orientation angle in the MD direction ($O$) with the similar topology of four, five, and one nodes in the input layer, hidden layer, and output layer, respectively, were developed. Moreover, another feed-forward back-propagation network with the same topology for secondary response (bending rigidity in the MD direction ($B$)) was built. Table 1 summarizes the value of hyperparameters that were undertaken for different networks.

**Table 1:** The parameter settings of different networks.

Parameter	Value
Normalization domain	[0.1, 0.9]
Data split ratio	27:3
Nodes in input layer	4
Nodes in hidden layer	5
Nodes in output layer	1
Transfer function of hidden layer	Hyperbolic tangent sigmoid
Transfer function of output layer	Pure linear
Learning algorithm	Levenberg*–*Marquardt algorithm
Learning rate	0.90
Momentum value	0.90
Number of epochs	1000
Number of runs	50
Network type	Feed-forward back-propagation
Software	MATLAB

During the development of the network, the best combination of hyper parameters was selected based on the procedure used by Refs.^55,56,64,65^. Moreover, the MATLAB toolbox was used to implement such networks.

### Genetic algorithm

There are various methods of optimization such as the golden section search (GSS) method, sequential quadratic programming (SQP) method, genetic algorithms (GAs), particle swarm optimization (PSO), ant colony optimization (ACO), and gray wolf optimizer (GWO), composite desirability function (CDF), and self-learning batch-to-batch optimization (SLBBO) method. All of the mentioned methods can be coupled with any function to perform optimization. In a condition where an ANN is applied, they are the only choices because differentiation cannot be employed^66–69^. Due to simplicity, GA was utilized to perform multi-objective optimization. The purpose of optimization was to achieve the maximum bending rigidity in the MD direction while keeping basis weight at a minimum. Thus, the cost function was constructed as Eq. (3).

3 $$Minimize: f=\sqrt{0\cdot 6({B}_{i}\left(\overrightarrow{x}\right)-0\cdot 9)+0\cdot 4({W}_{i}\left(\overrightarrow{v}\right)-0\cdot 1)} \forall \overrightarrow{x}\in {R}^{4\times 4} \& \overrightarrow{v}\in {R}^{4},$$

4 $${\overrightarrow{x}}_{i}=\left\{{W}_{i}\left(\overrightarrow{v}\right).{T}_{i}\left(\overrightarrow{v}\right).{C}_{i}\left(\overrightarrow{v}\right).{O}_{i}\left(\overrightarrow{v}\right)\right\} \& i=1.\dots .n,$$

5 $${\overrightarrow{v}}_{i}=\left\{{Y}_{i}.{L}_{i}.{P}_{i}.{F}_{i}\right\} \& i=1.\dots .n,$$

where $\overrightarrow{x}$ and $\overrightarrow{v}$ are vectors of the candidate solutions with four primary responses and vectors of the candidate solution with four independent parameters, respectively. It is worth mentioning that terms belonging to the normalized bending rigidity in the MD direction have more weight (0.6) than normalized basis weight (0.4) due to the importance of the parameters in end-use. Besides, the optimization was conducted in a way to obtain a fabric with the highest bending rigidity and lowest cost. In fact, nonwoven fabrics are sold based on fiber types and the basis weight. So, the basis weight acts as a function that lowers the total cost of fabric. During the optimization step, the constant settings as brought in Table 2 were used for GA under MATLAB software.

**Table 2:** Parameter settings of GA.

Parameter	Value
Population type	Double vector
Population size	100
Max generation	50
Creation function	Uniform
Scaling function	Rank
Selection function	Roulette
Elite count	1
Mutation function	Adapt feasible
Mutation fraction	0.5
Crossover function	Scattered
Crossover fraction	0.5
Migration direction/ interval	Forward/20% of population size
Migration fraction	0.90
Nonlinear constraints algorithm	Penalty
Cost limit	0

**Figure 1:**
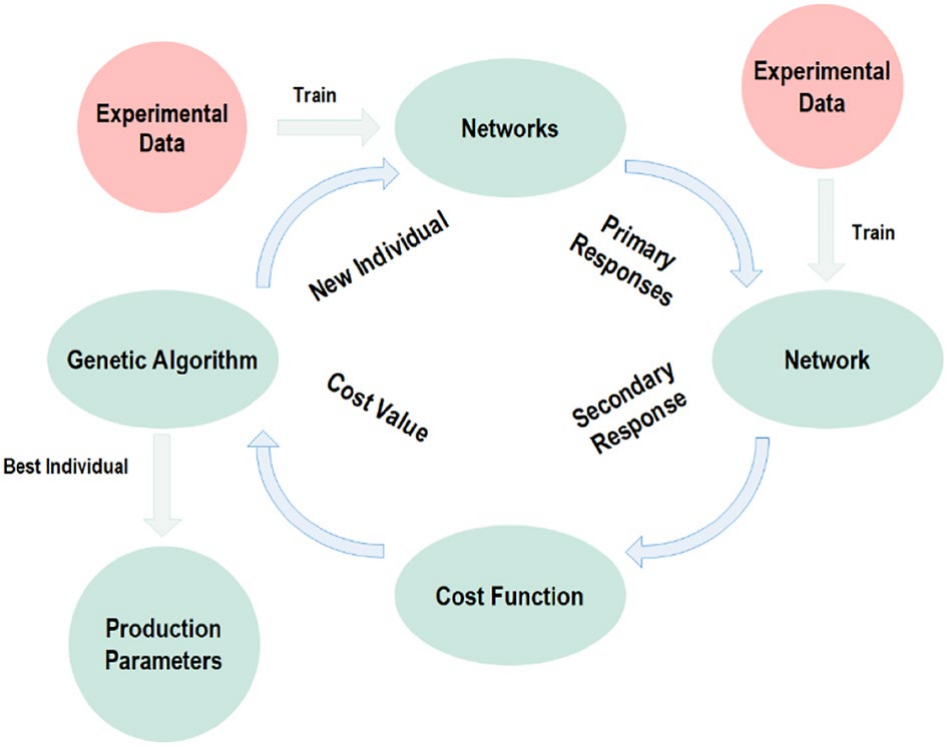
Flowchart of developed algorithm.

Figure 1 illustrates the whole algorithm developed in this work to seek out the combination of production parameters to obtain a nonwoven fabric with maximum bending rigidity and minimum basis weight. In this algorithm, after training networking for primary responses via experimental data and paralleling them together, a network is trained for secondary response using corresponding experimental data. In the next step, the cost function is formulated and coupled to the GA. When the cycle is complete, the GA creates a new individual and recalls the networks of primary responses. Then, the outputs are fed to network of secondary response. Finally, the cost function is evaluated and the stop criteria are checked by GA. Under the condition that stops criteria are satisfied, the best combination as the best individual is presented by GA.

## Results and discussion

### Statistical results

The mean value of primary and secondary responses for 30 samples is outlined in Table 3. According to the physics of the problem, when a sample possesses a higher basis weight (W), higher thickness (T), a higher number of couples (C), and higher fiber ordination distribution along the bending axis (O), more bending rigidity (B) will be achieved. Overall, a similar effect as the physics of the problem can be found in the data. However, there is some violations in data due to using different types of fiber.

**Table 3:** Mean value of primary and secondary responses.

Sample	Primary response								Secondary response	
	$W$(${\text{gsm}}$)		$T$(${\text{mm}}$)		$C$		$O$($\%$)		$B$(${\text{mN}}\cdot {\text{cm}}^{2}/{\text{cm}}$)	
	Act.	Norm.	Act.	Norm.	Act.	Norm.	Act.	Norm.	Act.	Norm.
1	53.00	0.7000	0.47	0.3933	1.39E+05	0.7273	61.26	0.4061	1.52	0.6677
2	38.00	0.2714	0.49	0.4467	7.87E+04	0.3048	63.29	0.5500	1.37	0.2806
3	60.00	0.9000	0.59	0.7133	1.63E+05	0.9000	67.94	0.8795	1.61	0.9000
4	32.00	0.1000	0.40	0.2067	6.84E+04	0.2322	62.01	0.4593	1.32	0.1516
5	56.00	0.7857	0.60	0.7400	1.40E+05	0.7347	68.23	0.9000	1.59	0.8484
6	45.00	0.3271	0.58	0.3933	6.59E+04	0.2025	62.65	0.5046	1.43	0.4355
7	38.00	0.2714	0.36	0.1000	9.31E+04	0.4060	59.17	0.2580	1.37	0.2806
8	55.00	0.7571	0.48	0.4200	1.46E+05	0.7805	62.69	0.5074	1.54	0.7194
9	40.00	0.3286	0.50	0.4733	8.55E+04	0.3525	64.56	0.6399	1.33	0.1774
10	47.00	0.5286	0.60	0.7400	9.84E+04	0.4430	67.93	0.8787	1.44	0.4613
11	42.00	0.3857	0.41	0.2333	9.99E+04	0.4535	59.52	0.2828	1.38	0.3065
12	43.00	0.4143	0.55	0.6067	7.79E+04	0.2990	61.01	0.3884	1.41	0.3839
13	42.00	0.3857	0.49	0.4467	8.35E+04	0.3382	59.84	0.3055	1.37	0.2806
14	55.00	0.7571	0.66	0.9000	8.66E+04	0.3603	65.08	0.6768	1.58	0.8226
15	43.00	0.4143	0.42	0.2600	1.02E+05	0.4698	58.38	0.2020	1.39	0.3323
16	36.00	0.2143	0.40	0.2067	7.51E+04	0.2796	57.81	0.1616	1.32	0.1516
17	48.00	0.5571	0.59	0.7133	1.04E+05	0.4852	67.88	0.8752	1.49	0.5903
18	38.00	0.2714	0.51	0.5000	7.56E+04	0.2829	62.31	0.4805	1.35	0.2290
19	38.00	0.2714	0.39	0.1800	8.59E+04	0.3553	59.91	0.3105	1.36	0.2548
20	40.00	0.3286	0.53	0.5533	5.70E+04	0.1519	61.19	0.4012	1.31	0.1258
21	42.00	0.3857	0.43	0.2867	9.52E+04	0.4206	60.23	0.3331	1.38	0.3065
22	56.00	0.7857	0.61	0.7667	1.38E+05	0.7184	65.19	0.6846	1.57	0.7968
23	34.00	0.1571	0.40	0.2067	6.70E+04	0.2223	56.94	0.1000	1.30	0.1000
24	54.00	0.7286	0.59	0.7133	9.35E+04	0.4086	64.23	0.6166	1.60	0.8742
25	35.00	0.1857	0.45	0.3400	5.14E+04	0.1126	59.98	0.3154	1.34	0.2032
26	34.00	0.1571	0.43	0.2867	7.18E+04	0.2563	62.07	0.4635	1.36	0.2548
27	54.00	0.7286	0.62	0.7933	8.89E+04	0.3765	64.77	0.6548	1.51	0.6419
28	42.00	0.3857	0.55	0.6067	6.06E+04	0.1770	61.79	0.4437	1.43	0.4355
29	34.00	0.1571	0.44	0.3133	4.96E+04	0.1000	60.23	0.3331	1.38	0.3065
30	38.00	0.2714	0.51	0.5000	5.35E+04	0.1270	60.55	0.3558	1.33	0.1774
Mean	43.565	0.430	0.498	0.468	8.98E+04	0.383	62.288	0.479	1.423	0.417
Minimum	60.000	0.900	0.660	0.900	1.63E+05	0.900	68.230	0.900	1.610	0.900
Maximum	32.000	0.100	0.360	0.100	4.96E+04	0.100	56.940	0.100	1.300	0.100
Variance	66.266	0.054	0.007	0.048	8.77E+08	0.043	9.552	0.048	0.010	0.064

**Figure 2:**
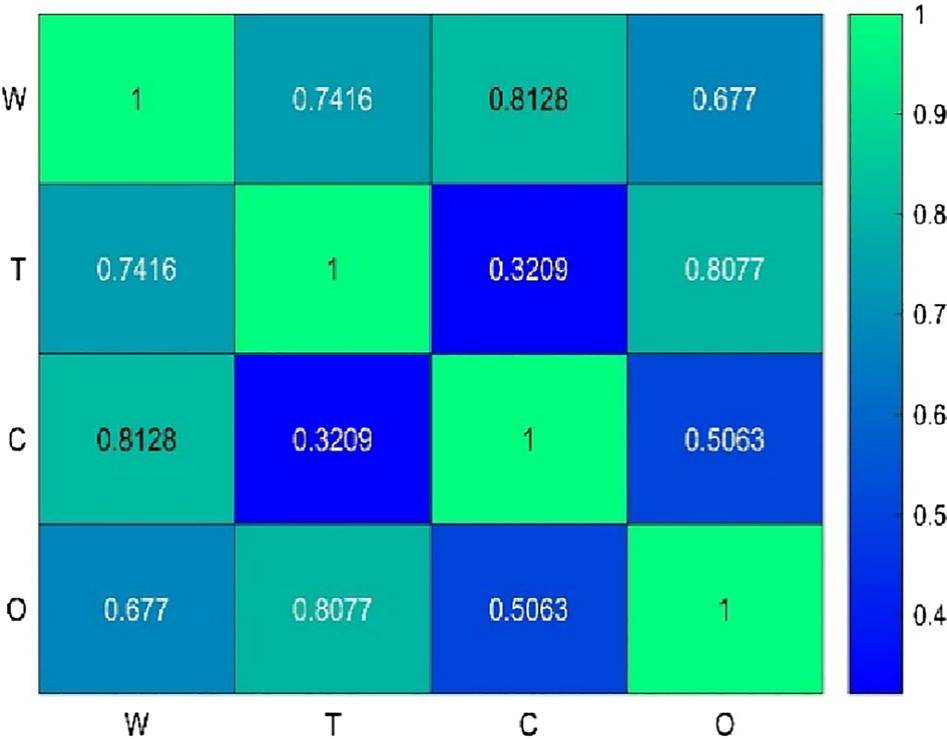
Pearson’s correlation heatmap matrix of primary responses.

Speaking of modeling via either RSM or ANN, it is favorable to avoid the redundancy of input variables of models. In fact, when there is a linear correlation between the input parameter of a model either positive or negative, multicollinearity has occurred which can make a negative impact on the accuracy of the model^51^. As can be seen in Fig. 2, low collinearity can be found between primary responses. Thus, elimination was not required for further steps. For elimination purposes, [− 0.9, 0.9] was considered a safe domain of correlation.

### RSM-based models

Table 4 provides an overview of the RSM-derived models created for both primary and secondary outcomes. The primary responses are determined by control parameters, while the input parameters for secondary responses are based on the primary outcomes.

**Table 4:** RSM-based models for different responses.

Basis weight	$W=0.2794-0.1584Y-0.3648L+0.1354P-0.1155F+0.0688{Y}^{2}+0.3349{L}^{2}-0.0451{P}^{2}+0.6789{F}^{2}-0.0384YL+0.1146YP+0.2349YF-0.1674LP+0.0926LF-0.0605PF$
Thickness	$T=0.2486+0.8531Y-0.6207L-0.7452P+0.8305F-0.6992{Y}^{2}+0.3482{L}^{2}+0.5842{P}^{2}-0.4033{F}^{2}-0.0034YL+0.2550YP+0.0590YF+0.0558LP+0.3168LF-0.1867PF$
Number of couples	$C=0.4434-1.5830Y-0.1516L+0.5493P-0.5159F+1.4799{Y}^{2}+0.2536{L}^{2}-0.3672{P}^{2}+0.8534{F}^{2}-0.0246YL+0.0094YP+0.2709YF-0.2748LP-0.0555LF+0.0794PF$
Fiber ordination	$O=0.1042+0.4054Y-0.4649L+0.3073P+0.5408F-0.1949{Y}^{2}+0.3871{L}^{2}-0.5006{P}^{2}-0.1637{F}^{2}+0.1401YL+0.3629YP+0.0924YF+0.0456LP-0.0299LF+0.0111PF$
Bending rigidity	$B=0.0970+0.1000W-1.1230T+0.3069C+1.1914O+0.7927{W}^{2}+4.0336{T}^{2}-2.5769{C}^{2}+0.7550{O}^{2}-5.5706WT+2.7244WC+5.2601WO+3.7215TC-4.6265TO-4.0236CO$

**Figure 3:**
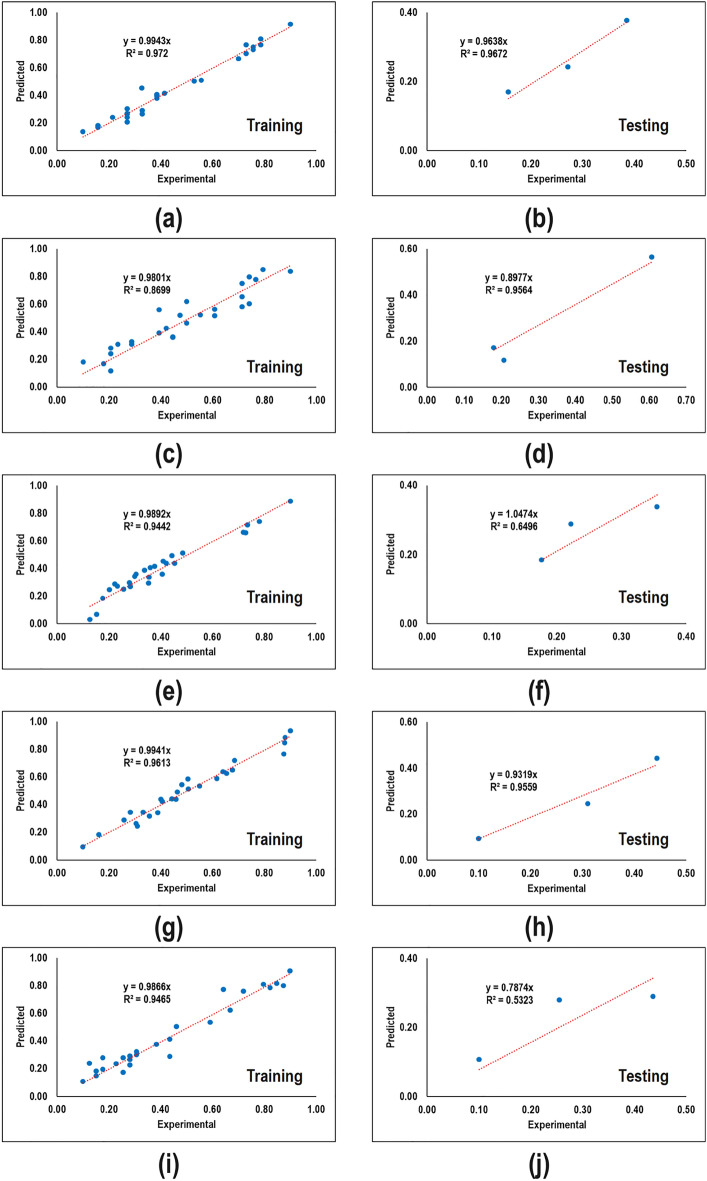
Performance of different RSM-based models during training and testing steps. (**a,b**) Basis weight, (**c,d**) thickness (**e,f**) number of couples, (**g,h**) orientation at MD direction, and (**i,j**) bending rigidity at MD direction.

Figure 3 compares the normalized value of predicted and experimental values for different responses. It can be seen that there are deviations between predicted and experimental values in all cases. This demonstrates that a relationship with a higher degree of nonlinearity lies among variables. Thus the developed RSM-based models are not reliable.

To compare the predictability of RSM-based models, the total goodness value which is the summation of the goodness value of training and testing steps multiplied by their corresponding data fraction that goodness value itself is the summation of the coefficient of determination and means the squared error is shown in Table 5. The ideal value of total goodness when the model is completely fit is 2. According to the information, the highest (1.9732) and lowest (1.8840) total goodness value was owned by basis weight and thickness, respectively.

**Table 5:** Quantitative comparison of RSM-based models.

Step	Parameter	$W$	$T$	$C$	$O$	$B$
Train	${R}^{2}$	0.9728	0.8836	0.9470	0.9629	0.9488
	$MSE$	0.0014	0.0057	0.0022	0.0017	0.0034
	$GF$	1.9714	1.8779	1.9448	1.9612	1.9454
Test	${R}^{2}$	0.9897	0.9422	0.9309	0.9764	0.8277
	$MSE$	0.0004	0.0033	0.0016	0.0014	0.0073
	$GF$	1.9893	1.9390	1.9293	1.9751	1.8205
$TGF$		1.9732	1.8840	1.9433	1.9626	1.9329

### ANN-based models

**Figure 4:**
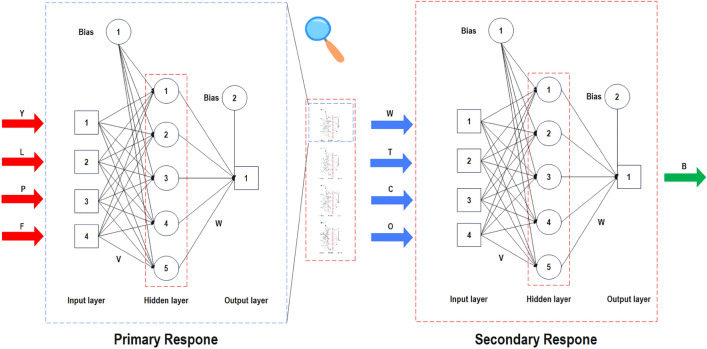
Topology of developed networks for primary and secondary responses.

Figure 4 shows the topology of networks trained for four primary responses ($W$, $T$, $C$, $O$) and a secondary response ($B$). As can be observed, four parallel feed-forward back-propagation network was trained in which production factors ($Y$, $L$, $P$, $F$) were the input parameters. Moreover, the output of four primary responses was fed to a network of secondary responses to result in bending rigidity. Surprisingly, all networks got four, five, and one node in their input, hidden, and output layers, respectively.

To provide information to build such networks mentioned above, weight and bias values are summarized in Tables 6, 7, 8 and 9 for primary responses and Table 10 for the secondary response.

**Table 6:** Weight and bias values of the ANN model of basis weight.

	Weight						Bias
W	− 2.5931	− 0.2373	2.5064	3.8635		1	3.9543
	− 1.7176	− 5.0689	2.0941	− 4.9099			4.9697
	− 4.2621	3.9785	1.9218	− 0.4416			2.3596
	2.7287	0.007	0.1988	− 4.4257			4.5816
	0.2689	− 0.6481	1.4332	1.6553			− 2.3114
V	0.3254	− 0.3124	− 0.3221	− 0.4319	0.4797	2	0.6467

**Table 7:** Weight and bias values of the ANN model of thickness.

	Weight						Bias
W	1.1472	− 2.7597	− 1.4793	− 2.1441		1	− 2.5485
	0.1537	− 0.5351	− 0.254	− 0.2458			− 0.4541
	− 2.4578	− 2.5091	− 2.6516	0.5677			− 0.0863
	− 0.7014	4.4312	1.4806	1.9327			− 0.8854
	− 0.069	− 2.0889	− 0.594	− 2.2806			2.8527
V	− 2.6357	6.6391	− 0.2136	1.008	− 1.0622	2	1.487

**Table 8:** Weight and bias values of the ANN model of number of couples.

	Weight						Bias	
W	− 0.1816	− 1.9113	− 5.5387	4.3123		1		3.3155
	− 1.4134	− 0.1952	− 2.8581	− 4.8203				3.8514
	− 0.4798	− 0.291	− 0.9354	− 0.7798				− 0.6108
	0.2379	− 0.164	− 0.5337	0.0113				0.981
	3.8122	− 0.033	− 0.9365	0.0866				3.3891
V	− 0.4645	− 0.9821	− 0.5517	4.2936	− 1.0392	2		− 2.0135

**Table 9:** Weight and bias values of the ANN model of orientation at MD direction.

	Weight						Bias
W	0.8766	− 0.5374	− 2.1887	− 1.0028		1	− 2.056
	− 5.2481	− 3.1908	2.4494	− 3.9471			2.2463
	− 2.815	2.2011	1.4567	− 2.9228			− 0.5336
	0.4235	− 0.8805	0.7614	− 0.237			2.5534
	− 0.3409	0.4816	0.1895	2.2887			2.9617
V	− 0.5389	− 0.4925	− 0.2316	1.3327	− 0.7154	2	− 0.8636

**Table 10:** Weight and bias values of the ANN model of bending rigidity at MD direction.

	Weight						Bias
W	− 0.9035	1.3757	3.1164	0.2036		1	2.1913
	9.9229	− 1.9466	− 5.5732	0.4868			− 1.3307
	− 4.8122	0.0223	2.6402	0.9709			1.0526
	− 2.9533	5.5797	− 0.8879	2.2523			− 2.4157
	− 1.3399	− 1.5651	1.1827	0.9054			1.1764
V	0.7025	2.9973	4.5958	− 1.2638	− 3.5121	2	− 0.1025

**Figure 5:**
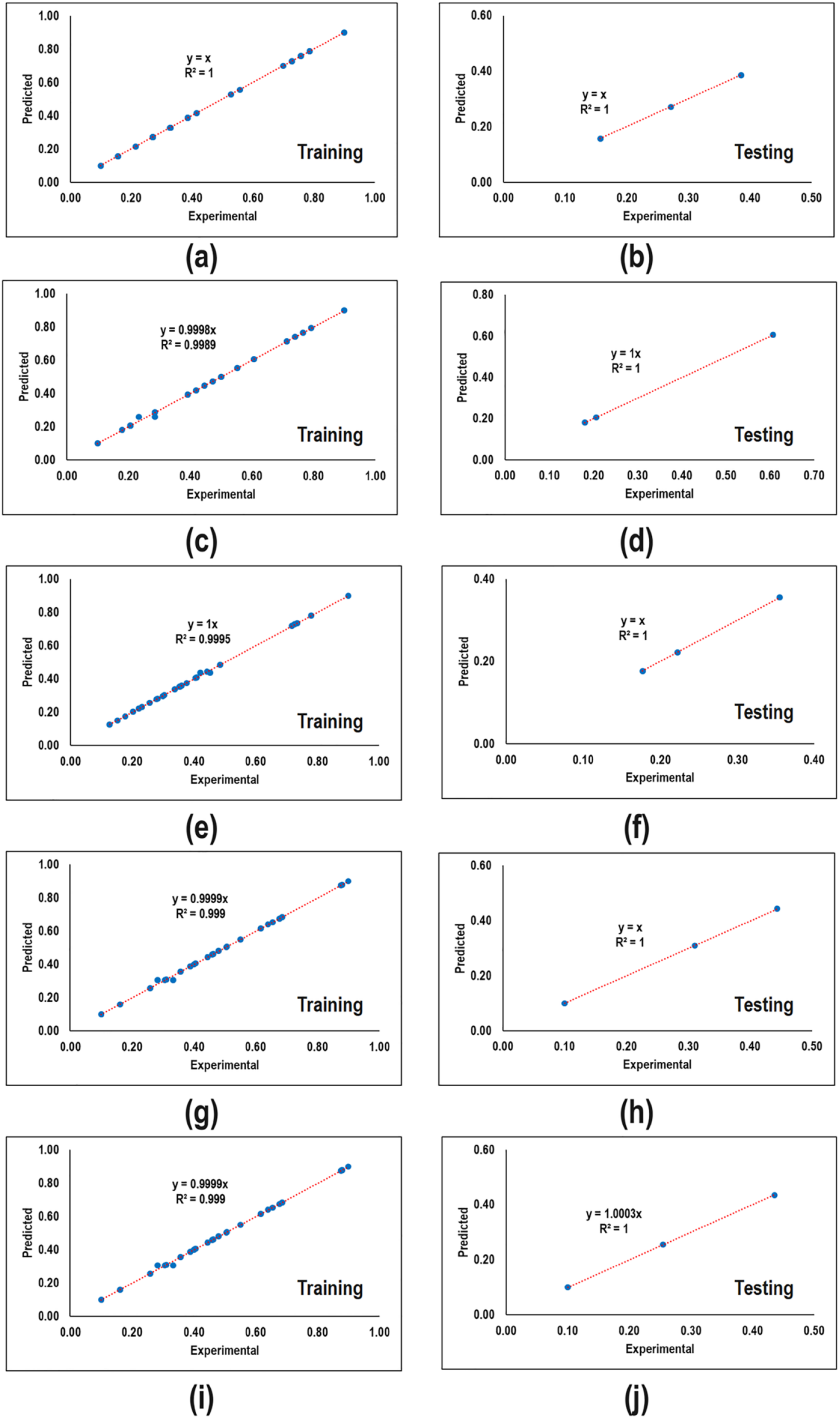
Performance of different ANN-based models during training and testing steps. (**a,b**) Basis weight, (**c,d**) thickness, (**e,f**) the number of couples, (**g,h**) orientation in the MD direction, and (**i,j**) bending rigidity in the MD direction.

To assess the performance of the network trained for various responses, predicted and experimental values were plotted as shown in Fig. 5. It is evident that the network was trained with a high performance of predictability. This means that they are able to perfectly map from input parameters to output ones. However, a few points of settlements were detected which are negligible.

To verify the better performance of networks compared to RSM-based models, a quantitative analysis of total goodness value is carried out. Table 11 demonstrates that all networks owned a total goodness value close to the ideal one (2). In fact, the high total goodness value indicates that the weight and bias values are adjusted perfectly.

**Table 11:** Quantitative comparison of ANN-based models.

Step	Parameter	$W$	$T$	$C$	$O$	$B$
Train	${R}^{2}$	1.0000	0.9990	0.9996	0.9991	1.000
	$MSE$	0.0000	0.0001	0.0000	0.0000	0.0000
	$GF$	2.0000	1.9989	1.9995	1.9990	2.0000
Test	${R}^{2}$	1.0000	1.0000	1.0000	1.0000	1.0000
	$MSE$	0.0000	0.0000	0.0000	0.0000	0.0000
	$GF$	2.0000	2.0000	2.0000	2.0000	2.0000
$TGF$		2.0000	1.9990	1.9996	1.9991	2.0000

**Figure 6:**
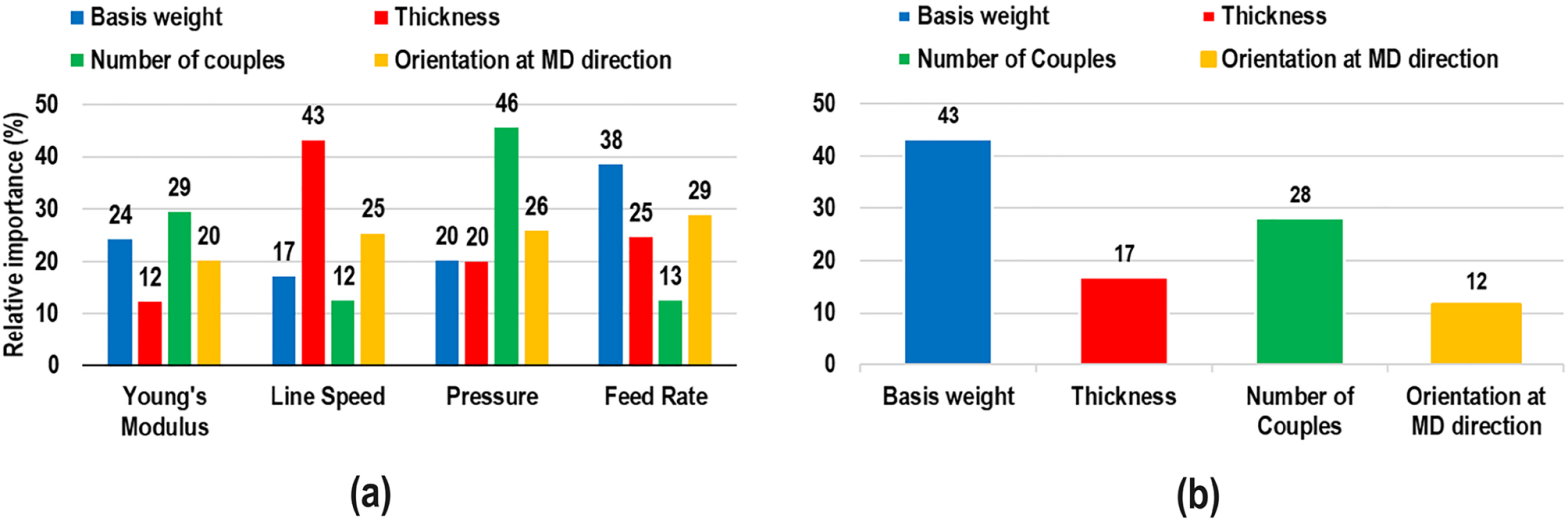
Sensitivity analysis of different networks, (**a**) primary, (**b**) secondary responses.

In order to specify the contribution of each input parameter in every network trained for prediction, Eq. (6) was used.

6 $${R}_{i}(\%)=\frac{\sum_{j=1}^{{n}_{H}}\left[\frac{\left|{V}_{ij}\right|\left|{W}_{j}\right|}{\sum_{l=1}^{{n}_{I}}\left|{V}_{lj}\right|}\right]}{\sum_{i=1}^{{n}_{I}}\left[\sum_{j=1}^{{n}_{H}}\left[\frac{\left|{V}_{ij}\right|\left|{W}_{j}\right|}{\sum_{l=1}^{{n}_{I}}\left|{V}_{lj}\right|}\right]\right]}\times 100,$$

where $V$ and $W$ are weights acting between the input layer and hidden layer and between the hidden layer and output layers, respectively. Parameters ${n}_{I}$ and ${n}_{H}$ indicate the number of nodes in the input layer and hidden layer, respectively. Figure 6 depict the results of sensitivity analysis conducted based on Eq. (6) for primary and secondary responses. According to Fig. 6a, it is clear that pressure (46%) makes the highest impact in the case of number of couples, while the most effecting node in network developed for thickness is line speed (43%). Turning to orientation at MD direction (29%) and basis weight (38%), it can be found that feed rate has the highest contribution in both cases. In the case of secondary response, the network is mostly effected by basis weight (43%) than other primary response as shown in Fig. 6b.

### Optimization results

**Figure 7:**
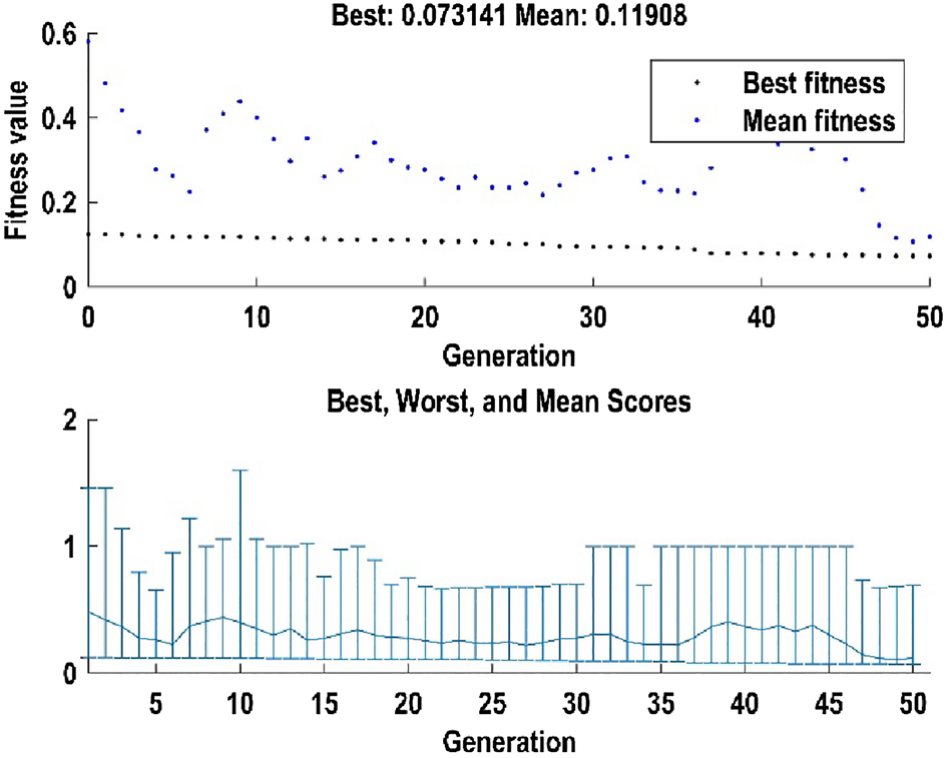
Performance of GA during optimization.

When optimization was performed via GA, the trend of reduction of cost value was observed as shown in Fig. 7. As can be seen that diversity of the constructed GA was high enough to avoid early convergence. Moreover, as more generation was created, the distance between best and mean value declined. At the end of the optimization, 0.0731 was the lowest cost value that resulted from Eq. (3). The way that GA reduced the cost value is by creating new combinations of production factors as new individuals and then calculating the primary and sequentially, secondary responses. Finally, when the cost value of the individuals is computed, the lowest one as the best individual is reported.

To consider the similarity and differences between optimized and optimum samples, production factors, primary responses, and secondary responses of them are summarized in Table 12. As it can be found that Young’s modulus of the optimized sample is lower than the optimum one while line speed and pressure were kept almost constant. Meanwhile, the feed rate of the optimized sample also went from 335 to 219.67 kg/h.

**Table 12:** Comparison between production factors, primary response and secondary response value of optimized and optimum samples.

Run	Value	$Y$ $({\text{GPa}})$	$L$ $(\text{m/min})$	$P$ $({\text{bar}})$	$F$ $(\text{kg/h})$	$W$ $({\text{gsm}})$	$T$ $({\text{mm}})$	$C$	$O$ $(\%)$	$B$ $({\text{mN}}{\text{cm}}^{2}/{\text{cm}})$	Cost value
Optimum	Predicted	0.6870	53.00	43.00	335.00	48.00	0.59	1.02e+05	67.88	1.49	0.375
	Exp.					48.00	0.59	1.04e+05	67.88	1.49	0.375
Optimized	Predicted	0.4195	53.91	42.43	219.67	36.03	0.31	4.46e+04	60.47	1.61	0.073
	Exp.					37.50	0.39	4.73e+04	62.82	1.43	0.373

Physically speaking, Young’s modulus of 0.4195 GPa indicate the viscose fiber which is more flexible than polyester (0.6870 GPa). As Young’s modulus of fiber lessens, its bending rigidity of it diminishes. So, at the constant line speed and pressure, more entanglement of fiber could be obtained. This will result in higher bending rigidity of the final product due to considerable cooperation of fiber under bending moment. In addition, the reduction of feed rate directly affects the basis weight. Besides, when a lower feed rate is set, the fiber has more opportunity to be entangled under water jet pressure. In other words, a reduction in feed rate increases the bending rigidity of nonwoven fabric. However, a low amount of feed rate is possibly having a negative effect on the bending rigidity of nonwoven fabric. To wrap it up, there is a feed rate that leads to the highest bending rigidity in which the negative effect and positive effects are balanced.

With regard to the primary response, it can be said that the basis weight due to the lower feed rate was significantly reduced from 48 to 37.5 gsm. Consequently, the thickness witnessed a reduction from 0.59 to 0.39 mm. Furthermore, the number of couples turned down from 1.04e+05 to 4.47e+04 because of the lower number of fiber acting within the fabric. In the case of fiber orientation disruption, a slight reduction was found (from 67.88 to 62.82%) which is the result of lowering the given time and applying force to fiber to orientate themselves along the machine direction according to the new combination of production factors. Ultimately, it can be seen that the bending rigidity of nonwoven fabric optimized by GA was close to the optimum sample while the basis weight was reduced. This brings about a cost value of the optimized sample lower than the optimum one.

In terms of perdition, it can be seen that prediction value regarding primary response was close to the experiment ones, whilst predicated cost value underwent an unacceptable difference. The driving force behind this could be the information data provided according to the design of the experiment. It is obvious that if a higher number of combinations were undertaken during the designing of the experiment, more information would be provided for the network. However, the high cost and time-consuming of taking more combinations limit the domain of work.

## Conclusions

By way of conclusion, in this work, a systematic study on mechanical and physical properties of spunlace nonwoven fabric was carried out to acquire information to train experimental-based methods such as artificial neural network and response surface methodology. Next, an optimization problem to achieve the highest bending rigidity and lowest basis weight of the nonwoven fabric was defined using a genetic algorithm. The results are as follows:

Response surface methodology not only is an effective procedure to collect data for modeling via experimental-based methods, but it also is capable of constructing a regression-based formula for prediction purposes. However, the performance of models developed by response surface methodology was not high enough. Thus, they have not been used in this work.

An artificial neural network as a modeling tool has the capability of creating experimental-based models with a high performance of predictability (R^2^ = 1). Its performance is much better than the response surface methodology based on the total goodness value obtained for different variables.

The genetic algorithm as an optimizer provides a combination of production factors (Young’s modulus of fiber of 0.4195 GPa, the line speed of 53.91 m/min, the average pressure of water jet 42.43 bar, and the feed rate of 219.67 kg/h) which leads to a sample with higher bending rigidity (1.43 mN ${\mathrm{cm}}^{2}$/cm) and lower basis weight (37.5 gsm) compared to the samples fabricated accordant to the design of experiment.

## Methods

### Materials and experimental design

To produce nonwoven fabric with different types of fibers, three kinds of fiber including polypropylene (PP), viscose, and polyester (PET) were sourced from Nikoogroup company. The Characteristics of fibers are summarized in Table 13.

**Table 13:** Characteristics of materials.

Material	Mean length (mm)	Mean diameter (μm)	Density ($\text{g/cm}$)
Polypropylene	40	18.90	0.91
Viscose	38	14.50	1.49
Polyester	38	13.60	1.38

To design an experiment, four independent parameters according to Table 14 namely called Young’s modulus with three levels (0.1820, 0.4195, 0.6872 ${\text{GPa}}$), line speed with three levels (50, 56, 60 $\text{m/min}$), the average pressure of water jet with three levels (38, 43, 48 ${\text{bar}}$) and feed rate with four levels (205, 270, 335, 400 $\text{kg/h}$) were selected as control variables.

**Table 14:** Independent parameters and their boundaries.

Parameter	Code	Unit	Level			
Young’s modulus	$Y$	${\text{GPa}}$	0.1820	0.4195	0.6872	
Line speed	$L$	$\text{m/min}$	50	56	60	
Pressure	$P$	${\text{bar}}$	38	43	48	
Feed rate	$F$	$\text{kg/h}$	205	270	335	400

Using Design-Expert 13 software to reduce the total number of samples, 30 combinations of independent parameters under the I-optimal type of central design of the experiment (CDOE) with a quadratic model based on randomized subtype without any blocks with 15 additional model points were determined as shown in Table 15. In addition, the training and testing datasets were shuffled by the randomized selection method with a split ratio of 27:3 for further steps.

**Table 15:** Combination of independent parameters.

Sample	Parameter								Dataset	
	$Y$(${\text{GPa}}$)		$L$($\text{m/min}$)		$P$(${\text{bar}}$)		$F$($\text{kg/h}$)		Train	Test
	Act.	Norm.	Act.	Norm.	Act.	Norm.	Act.	Norm.		
1	0.1820	0.1000	56	0.5800	43	0.5000	400	0.9000	+	−
2	0.6872	0.9000	50	0.1000	43	0.5000	205	0.1000	+	−
3	0.6872	0.9000	50	0.1000	48	0.9000	400	0.9000	+	−
4	0.6872	0.9000	60	0.9000	38	0.1000	205	0.1000	+	−
5	0.6872	0.9000	60	0.9000	48	0.9000	400	0.9000	+	−
6	0.4195	0.4761	53	0.3400	43	0.5000	335	0.6333	+	−
7	0.1820	0.1000	60	0.9000	43	0.5000	270	0.3667	+	−
8	0.1820	0.1000	50	0.1000	43	0.5000	400	0.9000	+	−
9	0.6872	0.9000	56	0.5800	48	0.9000	270	0.3667	−	+
10	0.6872	0.9000	60	0.9000	43	0.5000	335	0.6333	+	−
11	0.1820	0.1000	56	0.5800	43	0.5000	335	0.6333	+	−
12	0.1820	0.1000	53	0.3400	38	0.1000	335	0.6333	+	−
13	0.1820	0.1000	60	0.9000	48	0.9000	335	0.6333	+	−
14	0.4195	0.4761	50	0.1000	38	0.1000	400	0.9000	+	−
15	0.1820	0.1000	53	0.3400	48	0.9000	335	0.6333	+	−
16	0.1820	0.1000	50	0.1000	38	0.1000	205	0.1000	−	+
17	0.6872	0.9000	53	0.3400	43	0.5000	335	0.6333	+	−
18	0.6872	0.9000	50	0.1000	38	0.1000	270	0.3667	+	−
19	0.1820	0.1000	56	0.5800	43	0.5000	270	0.3667	+	−
20	0.4195	0.4761	60	0.9000	38	0.1000	270	0.3667	+	−
21	0.1820	0.1000	56	0.5800	43	0.5000	335	0.6333	+	−
22	0.6872	0.9000	56	0.5800	38	0.1000	400	0.9000	+	−
23	0.1820	0.1000	56	0.5800	38	0.1000	205	0.1000	+	−
24	0.4195	0.4761	56	0.5800	48	0.9000	400	0.9000	+	−
25	0.4195	0.4761	56	0.5800	43	0.5000	205	0.1000	+	−
26	0.6872	0.9000	56	0.5800	48	0.9000	205	0.1000	+	−
27	0.4195	0.4761	60	0.9000	38	0.1000	400	0.9000	+	−
28	0.4195	0.4761	50	0.1000	48	0.9000	270	0.3667	+	−
29	0.4195	0.4761	60	0.9000	48	0.9000	205	0.1000	−	+
30	0.4195	0.4761	50	0.1000	38	0.1000	205	0.1000	+	−

### Fabrication method

**Figure 8:**
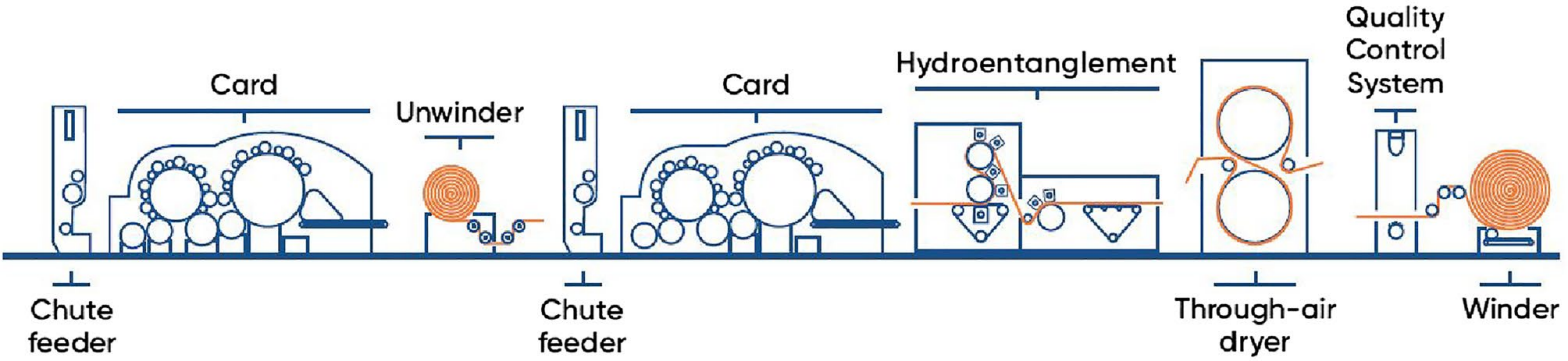
Schematic illustration of spunlace production line^70^.

Samples were fabricated via two sequential carding where an ANDRITZ Perfojet machine with a water jet was installed under a temperature of 32 °C and humidity of 40%. According to Table 15, through feeding different types of fibers with various values of line speed, pressure, and feed rate, a variety of samples were produced. Figure 8 demonstrates the schematic illustration of the production line.

### Characterization methods

**Figure 9:**
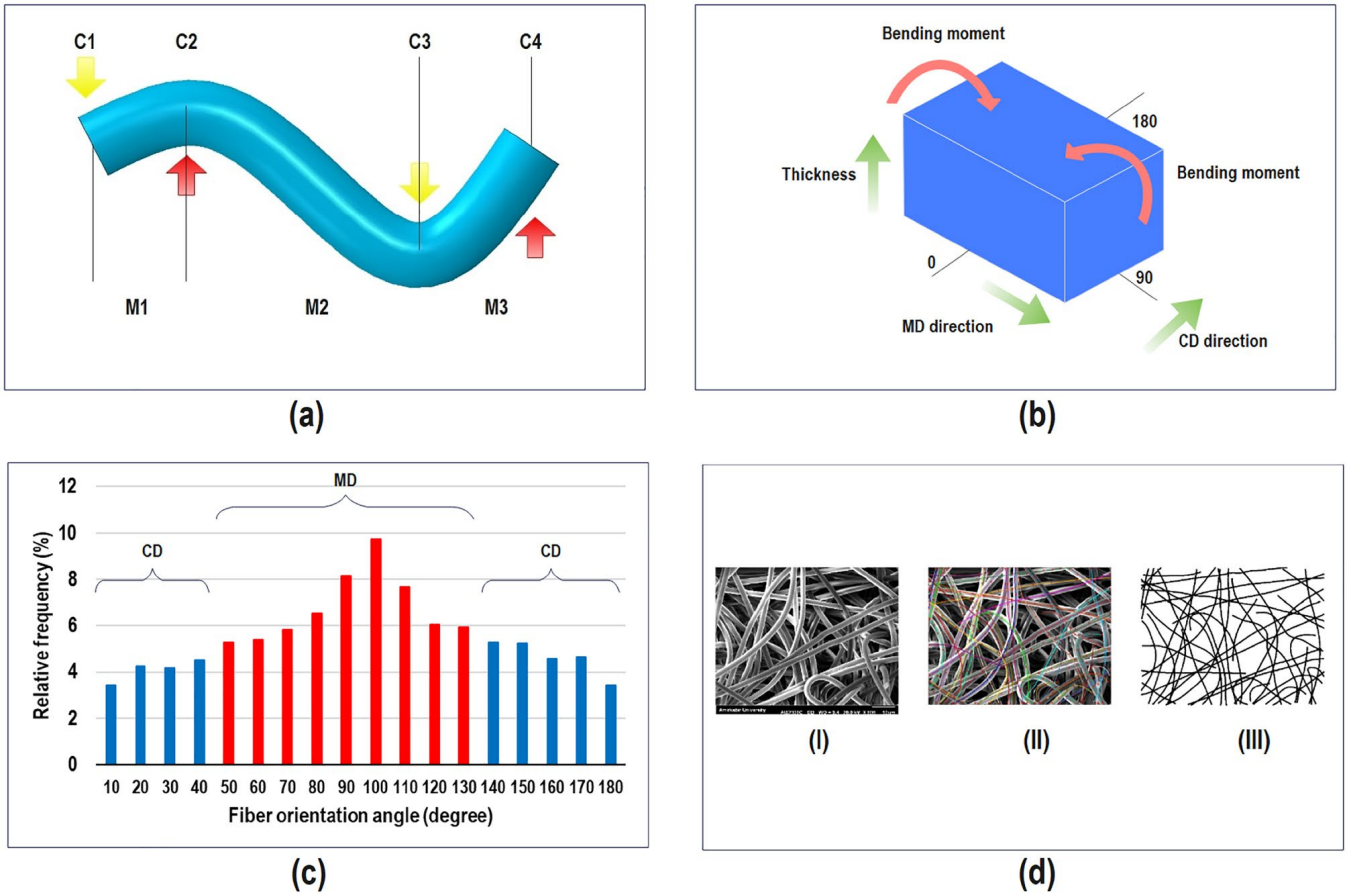
Fiber deformation under a multi-region couple of moments.

Fiber diameter was measured via Scanning electron microscopy (SEM, XL30-SFEG Philips, Japan) and utilizing Digimizer software in 100 points. Having determined the weight with a dimension of 10 $\times$ 10 ${\text{cm}}^{2}$ and 15 repetitions for each sample, the basis weight was specified. Relying on ASTM D 5729-97 test method, the thickness of samples was measured 15 times using Shirley digital thickness tester. Number of fiber was defined according to volume of fiber (${V}_{f}$) occupied in a fibrous layer with a volume of $V$ as below

7 $${n}_{f}=\left[\frac{{V}_{f}}{V}\right]=\left[\frac{4WA}{\pi {d}_{f}^{2}{\rho }_{f}\overline{{l }_{f}}}\right],$$

where $A$, ${d}_{f}$, and $\overline{{l }_{f}}$ refer to the effective area of the layer, the diameter of the fiber, and the mean length of the fiber, respectively. When a fibrous layer undergoes a bending moment, the fibers also witness bending moments. Meanwhile, fibers that are positioned on each other creating contact points, are building a multi-region couple of moments as shown in Fig. 9a. Considering the number of contact points and a number of couples, it can be seen that number of contact points is one unit less than the number of couples ($C$).

Thus, the formula derived by Komori and Makishima was used to estimate the number of fiber-to-fiber contact points and obtain the number of couples acting during the bending loading condition^71^.

8 $$C={n}_{c}-{n}_{f}={n}_{f}\left(\frac{2{d}_{f}{\overline{{l }_{f}}}^{2}{n}_{f}}{\pi V}-1\right).$$

Because of measuring the bending rigidity at zero angle, the percentage of fiber acting at [45,135]° is assumed to cooperate in the MD direction, and the percentage of fiber acting at [0, 45]° and [135, 180]° assumed to cooperate in the CD direction (Fig. 9b,c). To determine the distribution of fiber orientation angle, a simple program written based on the radon transformation function under MATLAB software was used (Fig. 9d). To ascertain Young’s modulus of the fibers, samples were pulled out from the structure and positioned onto a paper frame measuring 2 × 2 ${\text{cm}}^{2}$. Subsequently, the edges of the paper frame were trimmed using scissors. The sample was then subjected to tensile loading utilizing an (Instron 5566) testing apparatus at a stretching rate of 2 mm/min with a gauge length of 1 cm under an environmental condition with a temperature of 32 °C and humidity of 40%. To validate the findings, each sample underwent 10 repeated tests. The bending rigidity of the layer was assessed following the procedure^72^ trying 10 times to avoid noises. During the measurement of the bending rigidity of layers, samples with a dimension of 2.5 $\times$ 0.5 ${\text{cm}}^{2}$ witnessed a cyclic bending moment via weight of 0.20 g, and the slope of linear regions was taken.

## Data Availability

The data that support the findings of this study are available on request from the corresponding author.
